# Electroacupuncture alleviates sciatic nerve injury in sciatica rats by regulating BDNF and NGF levels, myelin sheath degradation, and autophagy

**DOI:** 10.1515/biol-2022-1035

**Published:** 2025-07-30

**Authors:** Zhengmao Liu, Qijuan Zhang, Xiaoli Zhang, Qing He, Yu Tian

**Affiliations:** Department of Rehabilitation Medicine, Wuhan Orthopaedics Hospital of Integrated Traditional Chinese and Western Medicine (The Affiliated Hospital of Wuhan Sports University), No. 279 Luoyu Road, Hongshan District, Wuhan, 430079, China; Wuhan Fiberhome Technical Service Co., Ltd, Wuhan, 430000, China

**Keywords:** electroacupuncture, sciatic nerve injury, nerve regeneration, autophagy

## Abstract

The aim of this study is to investigate the role of electroacupuncture in alleviating sciatic nerve injury. A rat model for sciatic nerve crush injury was established using clamping forceps, and then electroacupuncture at the “Zusanli” (ST36) and Huantiao” (GB30) acupoints was performed. The values of sciatic functional index (SFI), paw withdrawal latency (PWL), and paw withdrawal threshold (PWT) were evaluated. The mRNA expression of neurotrophic factors in nerve tissues was examined by RT-qPCR analysis. Morphometric analyses of transverse sections at the sciatic nerve distal to injury were performed 8 weeks post-injury. Immunofluorescence staining of sciatic nerve and western blotting were performed to measure the expression of myelin protein zero (MPZ) and autophagy markers. The experimental results displayed that the motor and sensory functions of sciatic nerve crush injury rats were restricted by pressure application. Electroacupuncture ameliorated sciatic nerve injury by increasing SFI, PWT, and PWL values in model rats during the recovery period. Electroacupuncture upregulated the mRNA expression of neurotrophic factors (brain-derived neurotrophic factor and nerve growth factor), increased the diameter of fibers and axon, and increased the thickness of myelin sheath after electroacupuncture. Electroacupuncture reduced MPZ and p62 expression while increasing LC3B and beclin-1 expression. In conclusion, electroacupuncture alleviates sciatic nerve injury by promoting nerve regeneration and autophagy.

## Graphical abstract

**Figure:**
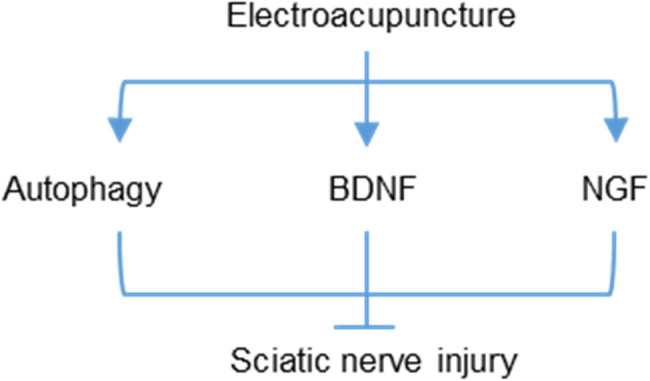

## 1 Introduction

The peripheral nervous system can be divided into cranial nerves, spinal nerves, and autonomic nerves [1]. Peripheral nerve injury (PNI) disturbs behavioral function and reduces life quality, becoming a challenge issue in rehabilitation medicine [2]. Unlike the central nervous system, the peripheral nervous system shows huge potential of self-repair and regeneration after PNI [3]. Nevertheless, complex injuries and massive loss of nerve tissues still call for therapeutic intervention to restore the functions. It is believed that the best way to regenerate functional nerves and restore their structures is autologous nerve transplantation by harvesting nerves from another site in the body. Unfortunately, the effects of autologous nerve grafts on PNI are not completely satisfactory [4]. Therefore, it is meaningful to explore more therapeutic methods for patients with PNI.

Animal experiments are preferred since it is difficult to study nerve repair and regeneration *in vitro* [5]. The most extensively used animal model for the investigation of peripheral nerve regeneration is the rat model for sciatic nerve crush injury because rat’s nerve trunk distribution is similar to that in human beings [6]. The rat model provides a nerve trunk in the middle of the thigh with enough space and length for surgical procedures, which allows investigators to apply a standard direct trauma in rats, thereby causing temporary or permanent changes in neurological functions and finally forming lesions similar to those in patients with PNI [7].

Electroacupuncture is one the most popular types of acupuncture that is widely used in China for its characteristics of effectiveness, safety, and few side effects [8]. It is acknowledged that electroacupuncture involves electrical current under precise-controlled parameters, which makes this technique more reproducible than manual acupuncture [9]. The efficiency of electroacupuncture has been demonstrated in multiple human diseases, such as knee osteoarthritis [10], depression [11], gut motility disorders [12], and reflex sympathetic dystrophy [13]. As known, electroacupuncture also presents a protective effect against nerve injury by mediating many biological mechanisms. For example, electroacupuncture mitigates cerebral ischemia/reperfusion injury by repressing autophagy via regulation of the SIRT1-FOXO1 pathway [14]. Electroacupuncture enhances nerve repair post PNI by targeting the microRNA-1b/brain-derived neurotrophic factor (BDNF) axis [15]. Moreover, electroacupuncture contributes to peripheral nerve regeneration after facial nerve crush injury [16].

The successful regeneration of peripheral nerves is dependent on the survival of neurons [17]. BDNF, a basic protein molecule, is vital for biological process involved in the nervous system, such as synaptic plasticity, apoptosis, survival, differentiation, migration, and proliferation [18]. Nerve growth factor (NGF) is an early regulator of nerve cell growth and is mainly synthesized by astrocytes and nerve cells. It can promote neurite outgrowth and nourish neurons. In addition, NGF displays crucial regulatory roles in the regeneration, growth, differentiation, and development of functional traits in central and peripheral neurons [19]. It has been reported that electroacupuncture has the potential to regulate the expression of both BDNF and NGF in cerebral ischemic rats [20], but the effects of electroacupuncture on BDNF and NGF in sciatic nerve injury have not been completely clarified yet.

Autophagy is a key cell process to maintain homeostasis through degradation of intracellular proteins and damaged organelles [21]. After the PNI, degradation of myelin sheath happens at the distal end of the injured nerve as tissue fragments begin to be broken down [22]. Autophagy of Schwann cells is important for the degradation and clearance of myelin debris after PNI [23]. The current study also explored myelin sheath degradation and autophagy in rat sciatic nerve tissues to evaluate the degree of sciatic nerve injury.

In the current study, the effect of electroacupuncture on sciatic nerve injury was explored by establishing a rat model. Then, the motor and sensory functions of rats as well as myelin sheath degradation and autophagy in rat sciatic nerve tissues were evaluated. Meanwhile, the expression of BDNF and NGF in the nerve tissues was detected to decipher the possible mechanisms underlying the action of electroacupuncture on neuropathic pain. This study may provide an effective therapeutic strategy for the treatment of patients with sciatic nerve injury.

## 2 Materials and methods

### 2.1 Animals and grouping

A total of 48 Sprague-Dawley rats (male, 190 ± 10 g, 5–6 week old) were purchased from Charles River Laboratories (Beijing, China) and caged separately in a room (18–22°C, 45% humidity) with artificial light and dark cycle (12:12). After acclimation to the environment for 7 days, the rats were used for animal experiments.

The FST toothless forceps (model: 13006-12) were used for the establishment of a rat model for sciatic nerve crush injury. Two positions, chosen for crush application, were located at one third of the distance from the hinge to the tip of the forceps (∼2.3 mm wide) and one third of the distance from the tip toward the hinge of the forceps (∼1.5 mm wide). These positions were pressed separately 1 notch, 2 notches, and 3 notches (the degrees of crush were one tooth, two teeth, and three teeth selected in the rachet in the handles) at either of the different positions. According to the location and the number of notches, 42 rats were randomly divided into 7 groups, with 6 individuals per group: control group, distal/proximal 1 notch group, distal/proximal 2 notches group, and distal/proximal 3 notches group. The rats in the control group were normal control rats. Despite the 42 rats for modeling, the rest 6 rats that we purchased were used as sham-operated ones for subsequent experiments.


**Ethical approval:** The research related to animal use has been complied with all the relevant national regulations and institutional policies for the care and use of animals, and has been approved by the Animal Ethics Committee of Wuhan Myhalic Biotechnology Co., Ltd (No. HLK-202302055; Date: 2023/2/10).

### 2.2 Animal model of sciatic nerve injury

The rat model for sciatic nerve crush injury was constructed as previously delineated [24]. The rats were intraperitoneally injected with 2% pentobarbital (30 mg/kg) for anesthetization. After the rats were plated in the prone position, the left sciatic nerve was exposed by making an incision on the mid-high of the left hind limb under aseptic conditions. The sciatic crush injury was created 10 mm above the bifurcation by the FST non-serrated clamp for 60 s, with the aim of obtaining good reproducibility of the sciatic nerve crush injury model. The sciatic nerve was then placed back under the muscle after being marked with a 11-0 non-invasive suture. All the surgical preparations were done by the same person to minimize the differences between rats. The rats in the sham group (*n* = 6) just underwent the exposure of left sciatic nerve without pressing.

### 2.3 Electroacupuncture

After modeling, the rats in distal/proximal 1 notch groups were divided into two groups (*n* = 6/group): Model group and Model + electroacupuncture (EA) group. The rats in the latter group were treated with electroacupuncture (20 min/day) for 8 weeks after modeling. The acupuncture needles were inserted into the acupoints, “Zusanli” (ST36) and Huantiao” (GB30) and connected to the negative and positive poles, respectively, and an intermittent wave at an electric current intensity that induced a slight muscle twitch was selected [25].

### 2.4 Motor function recovery analysis

The regeneration of sciatic nerve was evaluated by the sciatic functional index (SFI) at different time points: before modeling, 0 day after modeling, 3 days after modeling, as well as 2 weeks, 4 weeks, 6 weeks, and 8 weeks post-surgery. The SFI test was performed on a narrow corridor floor covered with clean white paper. All rats were trained to walk on the corridor floor before surgery. The hind feet of rat models were dipped in ink to record their footprints. The SFI of rats in each group was calculated by using the formula: SFI = 109.5 × (ETS [experimental toe spread] – NTS [normal toe spread])/NTS (normal toe spread) – 38.3 × (EPL [experimental print length] – NPL [normal print length])/NPL (normal print length) + 13.3 × ([EIT experimental intermediate toe spread] – NIT [normal intermediate toe spread])/NIT (normal intermediate toe spread) – 8.8 as previously reported [26,27]. The SFI value ranges from −100 to 0, with the complete dysfunction being −100 and the normal function being 0. The index score was −100 when no footprints were detected. In normal state, SFI value was calculated with the left footprint as experimental group, and right footprint normal group. The SFI value was calculated by an observer blinded to treatment allocation.

### 2.5 Paw withdrawal threshold (PWT)

According to the up-and-down method, PWT values of rat models were measured for mechanical sensitivity [28]. This test was performed in a noise-free room with constant temperature at different time points: before modeling, 3 days after modeling as well as 2 weeks, 4 weeks, 6 weeks, and 8 weeks post-surgery. A wire mesh (10 mm × 10 mm) was put on the bottom of a transparent plexiglass box (20 cm × 10 cm × 20 cm). Each rat was allowed to acclimatize to the environment of the box for half an hour. The center of the rat hind paw was stimulated by von Frey filaments (Yuyan bio, Shanghai, China) with a stimulating force (2, 4, 6, 8, 10, 15, and 26 g). The von Frey filaments with a force of 10 g were initially applied, and flinching or licking the paw were considered as positive reactions denoted by “X.” Subsequently, the filament was replaced with a neighboring filament of lower intensity; absence of response was denoted as “O.” A sequence was then established, with an “O” preceding an “X” assumed as the starting point. Six consecutive stimuli including the starting point were selected as key sequences for calculating the threshold of the 50% mechanical PWT. The stimulus duration was within 8 s, and the interval was 1 min for filaments. PWT refers to the minimum stimulative intensity causing a rat to trigger a contraction response. The experiment was measured in triplicates with at least 5 min intervals. The average value from three experiments was defined as PWT of rats.

### 2.6 Paw withdrawal latency (PWL)

The paw withdrawal response evoked by thermal hyperalgesia was assessed by the Hargreaves method [29]. This test was performed in a noise-free room with constant temperature at different time points: before modeling, 3 days after modeling, as well as 2 weeks, 4 weeks, 6 weeks, and 8 weeks post-surgery. Rats were placed on an evenly heated glass platform in the bottom of a transparent plexiglass box (20 cm × 10 cm × 20 cm). The plantar surface of the injured hind paw was focused by a radiant heat source in a quiet state. PWL was defined as the time period from the start of radiant heating to hind paw withdrawal [30]. After paw withdrawal, the heat source was turned off. A stimulus duration of 20 s was set to protect rats from injury. The heat source would be turned off exceeding 20 s even if a rat failed to make response. The mean value of three repeated measurements was defined as the PWL of rats. Two tests on the same rat were performed at a 10 min interval.

### 2.7 Morphometric analysis

Eight weeks after the surgery, the rats were anesthetized via intraperitoneal injection of 2% pentobarbital (30 mg/kg) and then sacrificed by cervical dislocation. Then, nerve tissues distal to the crush site of regenerated sciatic nerves in rats were harvested for morphometric analysis and polymerase chain reaction (PCR) analysis. The nerve samples were fixed with 2.5% glutaraldehyde and 1% osmium tetroxide, dehydrated in ethanol, resin-embedded, and sectioned into 1 μm slices. Then, the nerve tissues were stained with 1% toluidine blue (Sigma-Aldrich, St. Louis, USA). Morphometric parameters such as thickness of myelin sheath, number of myelinated fibers, and diameter of fibers in Figure 4 were calculated by ImageJ software (National Institutes of Health, Bethesda, USA).

### 2.8 Reverse transcription quantitative PCR (RT-qPCR) analysis

Total RNAs were extracted from nerve tissues using TRIzol reagent (Invitrogen, USA). PNI induces dramatic alterations in cellular composition reflected by the quantity and quality of RNAs, which makes the analysis of mRNA expression very complex. GAPDH was one of the housekeeping genes stably expressed under PNI conditions, which was thereof selected as the most confidential internal control gene by geNorm and NormFinder software. After RNA quantification, RNA was reverse transcribed to cDNA with the First-strand cDNA synthesis kit (Fermentas International Inc). PCR was performed with SYBR Green Master Mix (TargetMol, USA) on a real-time PCR system. The sequences of primers are listed in Table S1. Relative expression of mRNAs was determined by the 2^–ΔΔCt^ method [31].

### 2.9 Immunofluorescence staining

After the collection of the distal part of the injured sciatic nerves, the samples were cut into 6 μm sections and fixed with acetone for 15 min. The slides were washed with phosphate buffered saline (PBS) three times, blocked in 2% bovine serum albumin, and treated with 0.2% Triton X-100 for 1 h. After that, the slides were incubated with primary antibodies of anti-LC3B (ab192890, Abcam, UK) and anti-MPZ (ab39375, Abcam) at 4°C overnight. After PBS washing in triplicate, the sections were incubated with the secondary antibody followed by counterstaining with DAPI. A confocal fluorescent microscopy was used to capture the images. Scale bars were labelled using ImageJ software.

### 2.10 Western blotting

Sciatic nerve tissues were homogenized using radioimmunoprecipitation buffer supplemented with phosphatase and protease inhibitors and put on ice for 30 min. The solution was centrifuged at 12,000 g for 15 min at 4°C. After the isolation of supernatant, protein content was quantified using a bicinchoninic acid protein assay kit (Thermo Fisher Scientific, Waltham, USA). Next 20 μg of each sample was separated by electrophoresis on a 10% polyacrylamide gel and then transferred to a nitrocellulose membrane (Bioscience, Shanghai, China). Then, the membrane was blocked with 3% bovine serum albumin followed by incubation with primary antibodies at 4°C overnight. The primary antibodies were against anti-LC3Ⅱ (#4599, Cell signaling technology), anti-LC3Ⅰ (#43566, Cell signaling technology), anti-p62 (ab109012, Abcam, UK), anti-beclin-1 (ab302669, Abcam), anti-MPZ (ab39375, Abcam), and anti-β-actin (ab6267, Abcam). After that, the membrane was incubated with secondary antibody conjugated to horseradish peroxidase. Enhanced chemiluminescence kit (Thermo Fisher Scientific, Waltham, USA) was used to visualize the bands. The band intensity was quantified using the ImageJ software.

### 2.11 Statistical analysis

SPSS 25.0 software (SPSS Inc., Chicago, IL, USA) was employed to perform statistical analysis. Experimental data obtained from three biological and technical replications are expressed as the mean value ± standard error of the mean (SEM). Significant differences between two groups were compared using Student’s *t* test, and differences among three groups were evaluated using one-way analysis of variance (ANOVA) followed by Tukey *post hoc* test. The *p* value less than 0.05 was the threshold for statistical significance.

## 3 Results

### 3.1 Rat model for sciatic nerve injury was successfully established

**Figure 1:**
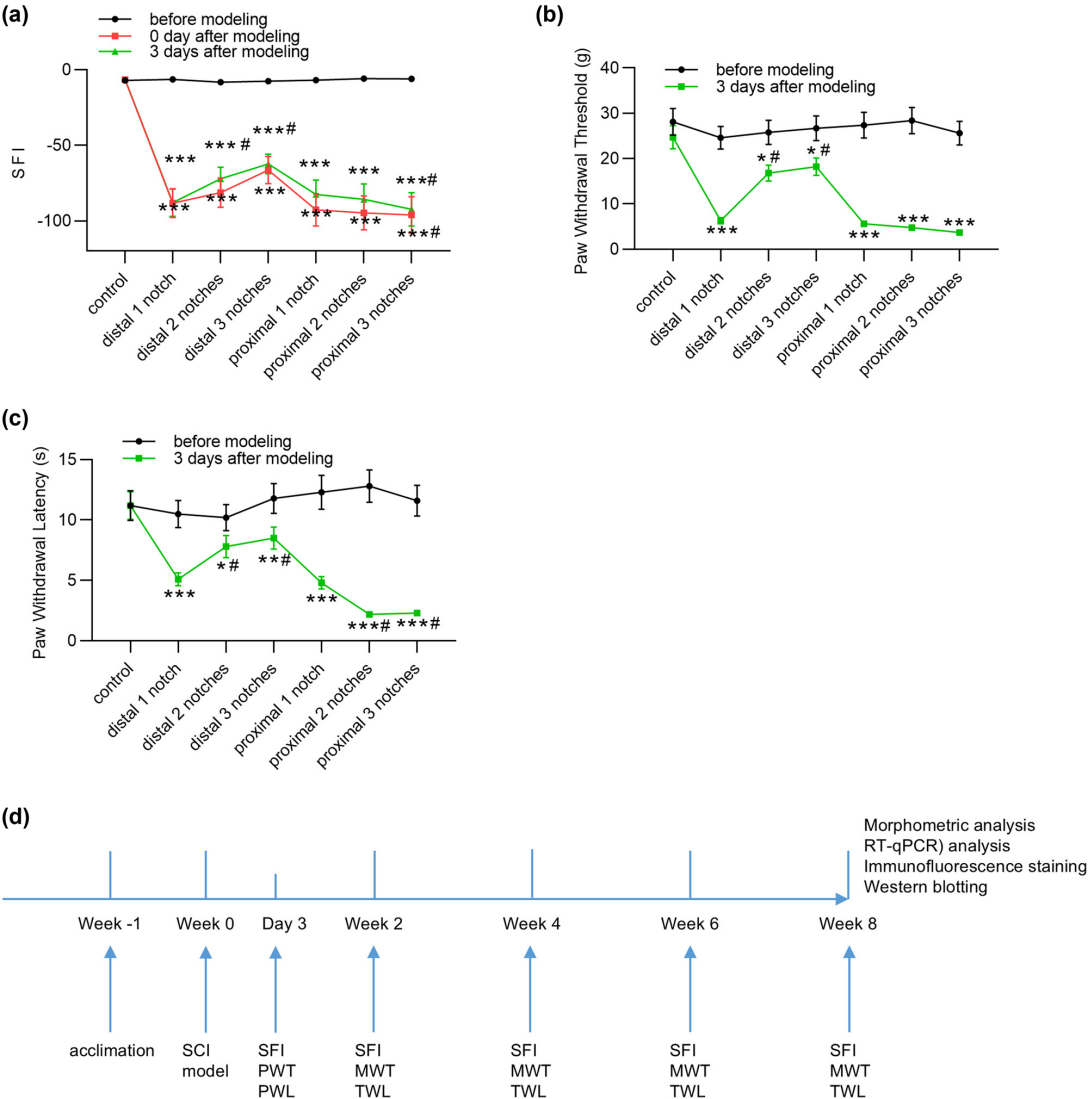
Establishment of a rat model for sciatic nerve injury. The sciatic crush injury was created 10 mm above the bifurcation by the FST non-serrated clamp for 60 s. The rats were randomly divided into 7 groups (*n* = 6/group): control group, distal/proximal 1 notch groups, distal/proximal 2 notches groups, and distal/proximal 3 notches groups. (a) SFI values were measured in the indicated groups before modeling, 0 day after modeling, and 3 days after modeling. (b) and (c) PWT and PWL in each group before modeling and 3 days after modeling. (d) The timeline of animal treatments. *n* = 6/group. *p < 0.05, **p < 0.01, ***p < 0.001 vs control group. ^#^ *p <* 0.05 vs distal 1 notch group or proximal 1 notch group.

SFI is a classical index of sciatic nerve function, which directly reflects the motor function post PNI. The SFI values of all rats were calculated and recorded before modeling, 0 day after modeling, and 3 days after modeling. As shown in Figure 1a, there was no significant difference in SFI values among all groups before modeling, but SFI values were significantly reduced in distal and proximal groups 0 day after modeling when compared to the values in sham group. Notably, there was no significant difference in SFI between the distal 1 notch group and the other groups except the proximal 3 notches group (Figure 1a). Three days post modeling, SFI values were markedly decreased in all injury groups relative to values in the sham group (Figure 1a). In addition, compared with the SFI value in distal 1 notch group, only values in distal 2 notches, distal 3 notches group, and proximal 3 notches groups were of significant difference (Figure 1a). PWT and PWL are two major means by which the sensory function of peripheral nerves can be evaluated. Mechanical allodynia and thermal hyperalgesia were assessed by PWT and PWL, respectively. Before modeling, PWT and PWL levels in sham rats of different groups showed no significant differences (Figure 1b and c). Three days after modeling, PWT and PWL levels were markedly downregulated in distal and proximal groups when compared to the levels before modeling (Figure 1b and c). Moreover, after modeling, PWT levels were notably elevated in the distal 2 and 3 notches groups compared to the PWT level in distal 1 notch group (Figure 1b). Compared with the PWL level in distal 1 notch group, a significant increase in PWL levels were discovered in distal 2 and 3 notches groups as well as a significant decrease in proximal 2 and 3 notches groups (Figure 1c). The alterations of SFI, PWT, and PWL values indicate the successful establishment of a rat model for sciatic nerve injury.

### 3.2 Electroacupuncture ameliorates sciatic nerve injury in model rats

**Figure 2:**
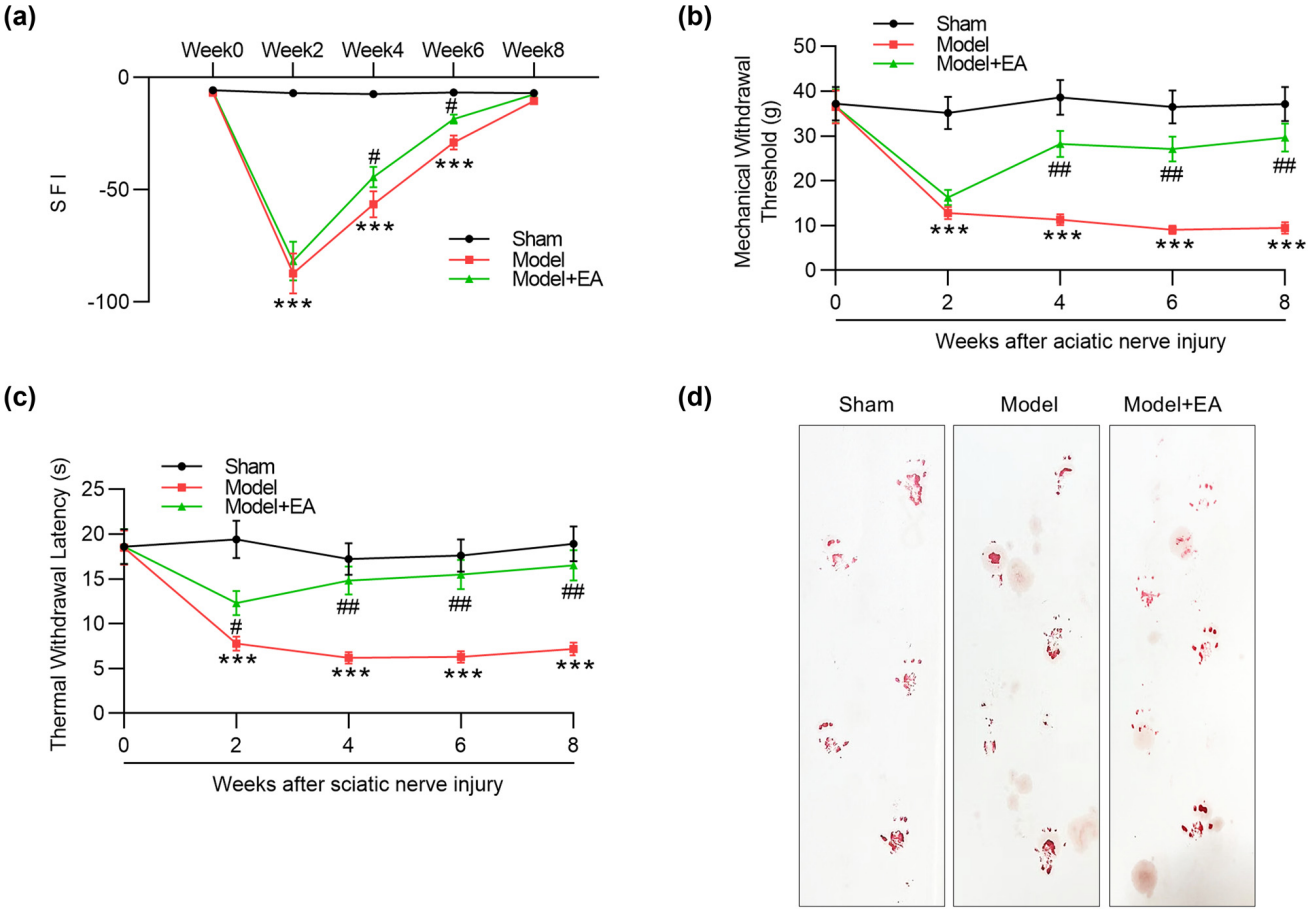
Electroacupuncture ameliorates sciatic nerve injury in model rats. (a) SFI values in the groups of Sham, Model, and Model + EA were recorded from week 0 to week 8 after sciatic nerve injury. (b) and (c) PWT and PWL in each group were recorded from week 0 to week 8 after sciatic nerve injury. (d) Ink recording of animal footprints in indicated groups. *n* = 6/group. The rats in distal/proximal 1 notch groups were used as models. ***p < 0.001 vs Sham group. ^#^ *p <* 0.05, ^##^ *p <* 0.01 vs Model grou*p*.

The rats in distal/proximal 1 notch groups were used as models. The SFI values in the model and model + EA groups were significantly decreased compared with values in sham-operated group, which reached the lowest point possibly at week 2 (Figure 2a), indicating the complete loss of function after nerve crush. The SFI values in two model groups showed an upward trend from week 2 to week 8 and reached pre-surgery baseline level at the last week. More importantly, electroacupuncture significantly increased the SFI values to −44.3 and −18.42 at week 4 and week 6 when compared to the values in the model group (−56.47 and −28.9) at the same time point (Figure 2a). The findings revealed that electroacupuncture led to faster nerve regeneration in model rats. In addition, at week 8, the SFI value in electroacupuncture group is closer to the pre-surgery baseline level than the Model group (Figure 2a). Then, we explored the effects of electroacupuncture on mechanical allodynia and thermal hyperalgesia in rat models by measuring PWT and PWL levels. Compared with the levels in sham-operated rats, PWT and PWL levels were significantly reduced in the model group, and electroacupuncture treatment reversed the suppressed PWT and PWL levels in model rats (Figure 2b and c). Moreover, normal animal footprints were observed in sham group, while abnormal footprints were observed in model and model + EA groups (Figure 2d).

### 3.3 Electroacupuncture increases levels of neurotrophic factors in sciatic nerve injury rats

**Figure 3:**
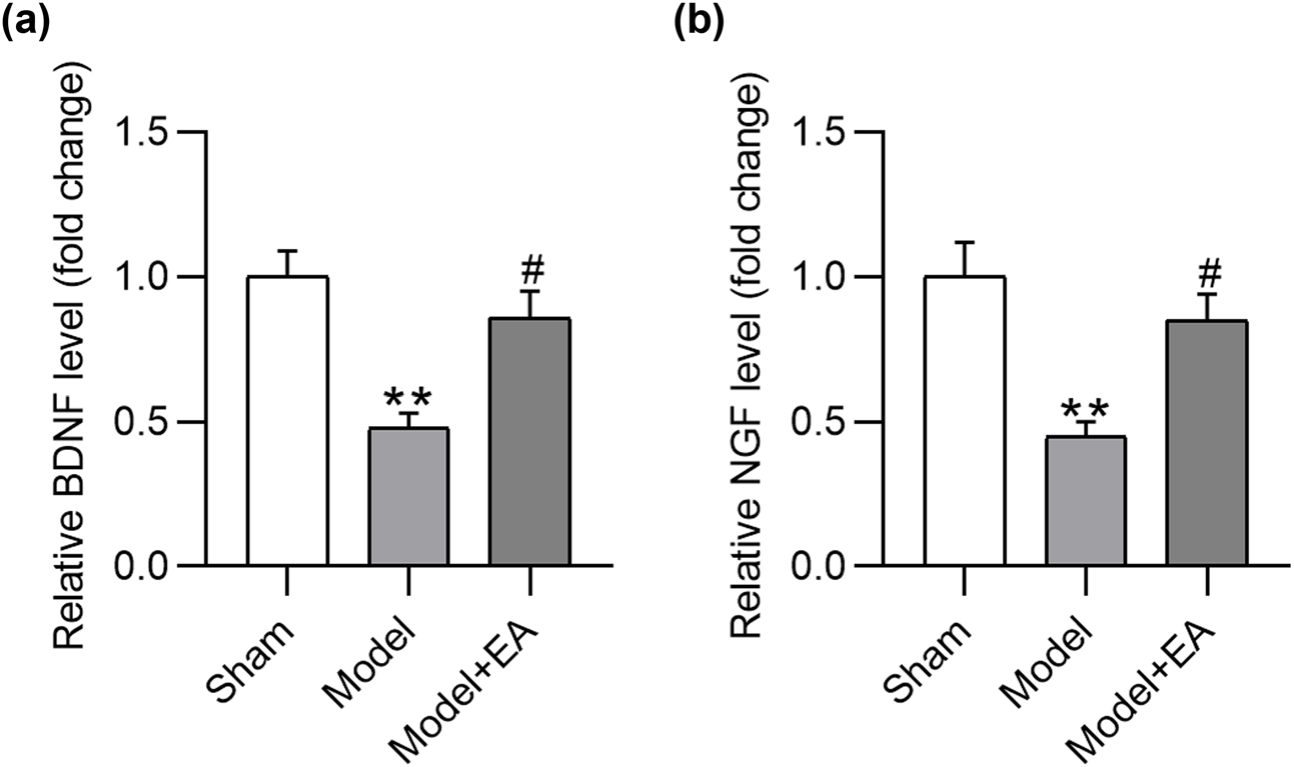
Electroacupuncture increases levels of neurotrophic factors in sciatic nerve injury rats. (a) and (b) The mRNA expression of BDNF and NGF in nerve tissues of Sham group, Model group, and Model + EA group was examined by RT-qPCR analysis. *n* = 6/group. **p < 0.01 vs Sham group. ^#^ *p <* 0.05 vs Model group.

Neurotrophic factors such as BDNF and NGF can promote the survival and function of nerve cells throughout the process of nerve regeneration. Therefore, the mRNA expression of BDNF and NGF in nerve samples were examined by RT-qPCR after sciatic nerve injury (at week 8). The results implied that BDNF and NGF mRNA expression were significantly downregulated in the model group relative to expression levels in the sham group, and the inhibitory impact on both neurotrophic genes was countervailed by electroacupuncture treatment (Figure 3a and b).

### 3.4 Quantitative morphological analysis of regenerated nerves

As shown in Table 1, at the end of week 8, there was an increase in the number of myelinated fibers and a decrease in diameter of fibers and axons as well as myelin sheath thickness in the model group relative to those in the sham group. However, the number of myelinated fibers was further increased in model rats receiving electroacupuncture treatment (Table 1). Moreover, the reduced values in diameter of fibers and axons as well as myelin sheath thickness were regained after electroacupuncture treatment (Table 1).

**Table 1:** Quantitative morphological analysis of regenerated nerves. Morphometric analyses of transverse sections at the sciatic nerve distal to injury for each of the experimental groups 8 weeks post-injury

Groups	Number of myelinated fibers	Diameter of fibers (µm)	Diameter of axon (µm)	Thickness of myelin sheath
Sham	8924.74 ± 91.36^a^	5.86 ± 0.13^a^	4.36 ± 0.06^a^	0.84 ± 0.02^b^
Model	9088.52 ± 90.15	5.5 ± 0.05	4.17 ± 0.06	0.62 ± 0.02
Model + EA	10247.36 ± 41.58^c^	5.77 ± 0.08^a^	4.33 ± 0.06^a^	0.74 ± 0.02^b^

Morphometric analyses of transverse sections at the sciatic nerve distal to injury for each of the experimental groups 3 weeks post-injury. Values shown as mean value ± SEM ^a^ *P* < 0.05, ^b^ *P* < 0.01, and ^c^ *P* < 0.001 vs control group.

### 3.5 Electroacupuncture accelerates the degradation of myelin sheath and promotes autophagy after sciatic nerve injury

**Figure 4:**
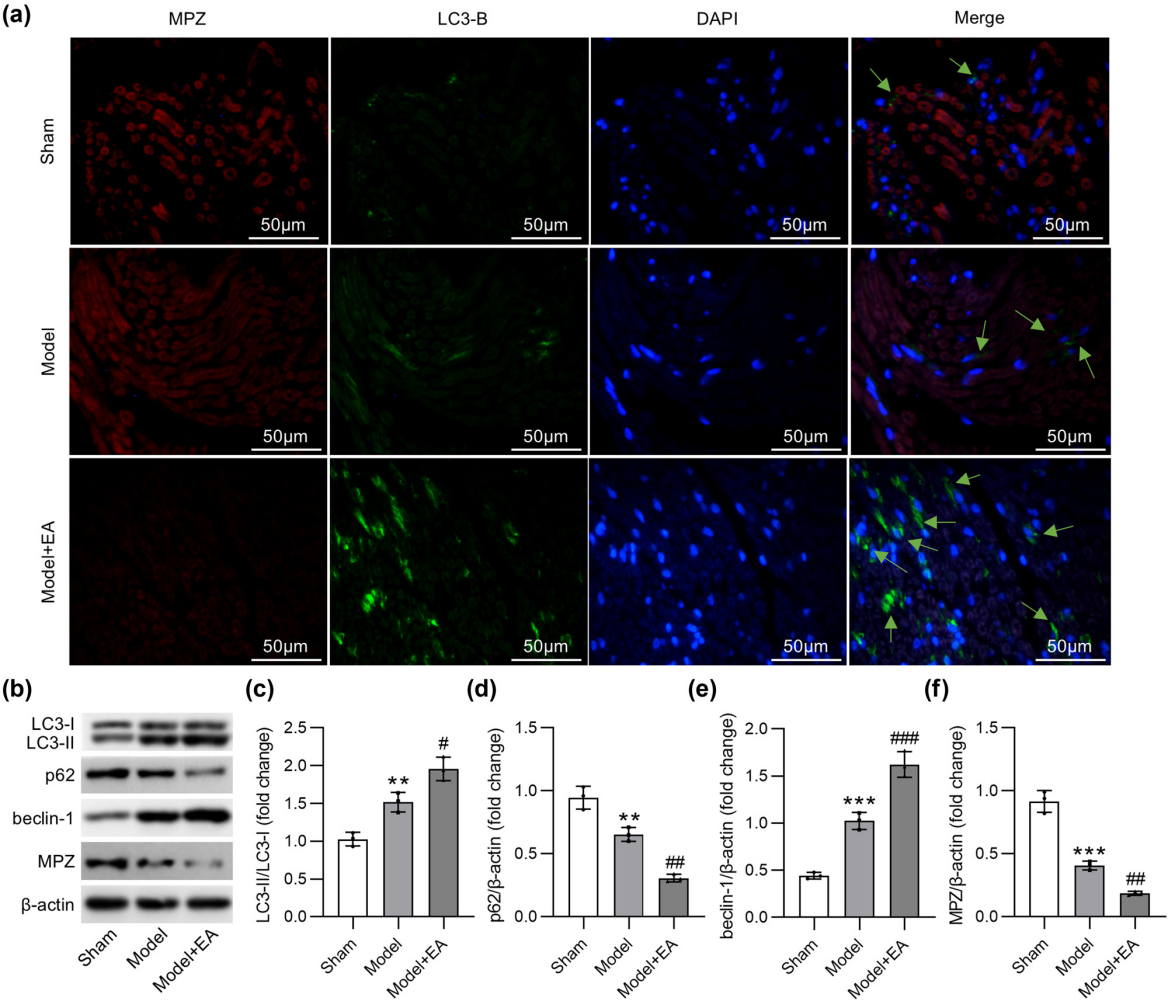
Electroacupuncture accelerates the degradation of myelin sheath and promotes autophagy after sciatic nerve injury. (a) Immunofluorescence staining of sciatic nerves in Sham, Model, and Model + EA groups was conducted to measure the protein level of autophagic marker LC3B (shown by green arrows) and myelin specific protein MPZ. (b)–(f) Western blotting was performed to quantify. (c) the ratio of LC3Ⅱ/LC3Ⅰ, (d) beclin-1, (e) p62, and (f) MPZ protein levels. **p < 0.01, ***p < 0.001 vs Sham group. ^#^ *p <* 0.05, ^##^ *p <* 0.01 vs Model group.

Eight weeks after surgery, rats were sacrificed, and sciatic nerve tissues were collected for Western blotting and immunofluorescence staining. Immunofluorescence staining of the sciatic nerves was performed to measure the expression of myelin protein zero (MPZ) and the autophagy marker LC3B in sciatic nerve. As shown in Figure 4a, MPZ expression was markedly reduced in the distal end of the damaged nerves compared to that in the sham group. Electroacupuncture treatment enhanced the reduction in MPZ expression (Figure 4a), suggesting the promoting role of electroacupuncture toward the degradation of myelin sheath. LC3B expression (shown by green arrows) was slightly increased in the damaged nerves of the model group compared to that in nerves of sham-operated rats, and it was abundantly expressed in rats that received electroacupuncture (Figure 4a). The results indicated that electroacupuncture could promote autophagy process to facilitate the recovery of injured nerves. Western blotting was conducted to measure protein levels of MPZ and autophagic factors. LC3Ⅱ/LC3Ⅰ ratio and beclin-1 level were increased while p62 expression was decreased in the nerve-injured group, and the changes were enhanced in rats that received electroacupuncture (Figure 4b–e). Consistent with the results of immunofluorescence staining, MPZ protein level was reduced in rats with sciatic nerve injury, and the reduction was aggravated after electroacupuncture treatment (Figure 4f). Overall, electroacupuncture accelerates the degradation of myelin sheath and promotes autophagy to facilitate the recovery of sciatic nerve injury.

## 4 Discussion

The leading challenges for neurological function restoration are neuroprotection, repair, and regeneration [32]. The regeneration potential of nerve fibers after injury can be presented as the *de novo* expression or elevation of various molecules in the distal nerve fiber tracts [33]. Neuronal response is also relevant to the expression of growth-stimulating substances, transcription factors, neurotrophic factors (e.g., BDNF and NGF), and extracellular matrix component [16]. Electroacupuncture is a kind of acupuncture widely used in clinical practice [14]. The present study intended to investigate the effect of electroacupuncture on nerve regeneration in a rat model of sciatic nerve crush. We found that electroacupuncture promoted sciatic nerve regeneration post sciatic nerve injury and upregulated BDNF and NGF expression in nerve tissue samples. In addition, electroacupuncture promotes the degradation of myelin sheath and accelerates autophagy in sciatic nerve tissues of model rats.

The SFI value, representing a classical indicator of motor function, is widely utilized to explore the function of nerves following PNI [34]. The obvious limitation in motor function on the injured side was observed in the sciatic nerve clamp injury model. Given that rats experienced pain within the acute period of sciatic nerve crush injury, the rats showed lameness on 0 day after modeling, which suggested the temporary complete loss of motor function. On the third day after modeling, SFI measurement results manifested that the walking ability of rats was gradually weakened as the clamp force value increased during the modeling process. The injured limb was completely off the ground or dragged when the pressure increased to the distal 1 notch pressure, suggesting the entire loss of motor function. PWT and PWL are two primary ethological indices for the quantitative evaluation of sensory function recovery [28], and both PWT and PWL values are markedly reduced in sciatic nerve injury rats.

Electrical stimulation can contribute to peripheral nerve regeneration and target nerve regeneration [35]. Electroacupuncture stimulation at ST36 and GB30 acupoints has been validated to ameliorate muscle atrophy induced by sciatic nerve injury [36]. Previous studies demonstrate that acupuncture at the GB30 acupoint enhances the regeneration of axons in damaged sciatic nerves and promotes motor recovery [37]. Stimulation via electroacupuncture relieves neuroedema, improves muscle nutrition and accelerates the speed of functional recovery in rats with sciatic nerve injury [38]. The insertion of acupuncture needles to rats at GB30 and ST36 acupoints accelerates the recovery of the injured sciatic nerves post sciatic nerve crush injury [39]. According to our results, even injured rats without any treatment had some nerve self-healing ability. However, electroacupuncture treatment accelerates the functional recovery of the sciatic nerves, as manifested by the upregulated values of SFI, PWT, and PWL as well as the increases in number of myelinated fibers, diameter of fibers and axons and myelin sheath thickness in sciatica rats.

BDNF and NGF, which are neurotrophic factors, exert a crucial function in both the central and peripheral nervous systems [40]. Neuronal activity can regulate the release and synthesis of BDNF, and BDNF in turn contributes to trophic effects including the initiation and stabilization of synapses [41]. NGF, mainly synthesized by astrocytes and nerve cells, can protect neurons by reducing the toxicity of excitatory amino acids and facilitate the clearance of free radicals [42]. Some studies reveal the promoting role of BDNF and NGF in the repair of nervous system injury by promoting the differentiation and regeneration of injured neurons and contributing to neuronal survival, growth, and apoptosis inhibition following ischemia [43,44]. It has been reported that electroacupuncture at “Changqiang” (GV 1) acupoint upregulates the expression of BDNF and NGF in rats with acute spinal cord injury [45]. Electroacupuncture at GB30 and ST36 acupoints benefits the repairment of PNI-induced nerve injury by regulating BDNF [15], and acupuncture at GB30 acupoint also improves the pathological alterations and the damaged function of sciatic nerves in sciatica rats undergoing sciatic nerve cut off by up-regulating NGF [46]. Consistently, our study showed that electroacupuncture at GB30 and ST36 acupoints upregulated the mRNA expression of BDNF and NGF in sciatica rats.

MPZ has been widely utilized for the labeling of myelin sheaths surrounding myelinated nerve fibers [47]. On one hand, exercise and physiotherapy have been demonstrated to exert beneficial effects in maintaining MPZ levels [48]. Conversely, the expression of MPZ has been observed to significantly decrease following sciatic nerve crush injury [27]. This degradation of myelin sheath protein is believed to occur through autophagy [27]. In the current research, autophagy-mediated downregulation of MPZ was also observed post sciatic nerve injury. After PNI, the degradation of myelin sheath occurs at the distal end of the injured nerve as tissue fragments begin to break down [49]. The autophagy process effectively promoted clearance of myelin debris following PNI [50]. Our study demonstrated that EA significantly facilitated cell autophagy to ameliorate nerve injury. In conclusion, electroacupuncture at “Zusanli” and “Huantiao” acupoints ameliorates clamping forceps-induced sciatic nerve injury by promoting nerve regeneration, upregulating the expression of BDNF and NGF, and promoting autophagy and myelin sheath degradation in sciatic nerve tissues, suggesting that electroacupuncture might become a therapeutic operation for clinical treatment of PNI. However, the best stimulation frequency for sciatic nerve injury remains to be further explored, and the mechanisms require further investigation as well.

## Supplementary Material

Supplementary Table
