# Supplementary material for: Electroacupuncture alleviates sciatic nerve injury in sciatica rats by regulating BDNF and NGF levels, myelin sheath degradation, and autophagy
Source: Open Life Sci. 2025 Jul 30;20(1):20221035. doi: 10.1515/biol-2022-1035 (PMC12317652; doi:10.1515/biol-2022-1035)
Supplement: Supplementary Table [file biol-2022-1035-sm.pdf]

Supplementary material

Table S1: Sequences of primers used for reverse transcription-quantitative PCR

Gene	Sequence (5′→3′)
BDNF	Forward: 5′-GGTCACAGTCCTGGAGAAAG-3′
	Reverse: 5′-GTCTATCCTTATGAACCGCC-3′
NGF	Forward: 5′-ACCTCTTCGGACACTCTGGA-3′
	Reverse: 5′-GTCCGTGGCTGTGGTCTTAT-3′
GAPDH	Forward: 5′-CCACGGCAAGTTCAACGGCACAGT-3′
	Reverse: 5′-CAGCGGAAGGGGCGGAGATGAT-3′
